# Supplementary material for: Factors Required for Adhesion of Salmonella enterica Serovar Typhimurium to Lactuca sativa (Lettuce)
Source: Microbiol Spectr. 2022 Dec 19;11(1):e03436-22. doi: 10.1128/spectrum.03436-22 (PMC9927257; doi:10.1128/spectrum.03436-22)
Supplement: Supplemental file 1 — Supplemental material. Download spectrum.03436-22-s0001.pdf, PDF file, 1.1 MB [file spectrum.03436-22-s0001.pdf]

Factors required for adhesion of *Salmonella enterica* serovar Typhimurium to *Lactuca sativa* (lettuce)

Laura Elpers[1], Lena Lüken[1], Fabio Lange[1], Michael Hensel[1, 2]

[1]Abt. Mikrobiologie, [2]CellNanOs – Center for Cellular Nanoanalytics

Universität Osnabrück, Osnabrück, Germany

Supplementary Materials

Suppl. Table

Table S1. Oligonucleotides used in this study

Designation	Sequence (3´ -> 5´)	Purpose
Vf-MisL-78	GTTTATGGCAATGGCCTCACGCTTAATGGG	SDM of p4403
Vf-MisL-171	TATAACTCACTGGGCCAGGGTTG	SDM of p4403
Vf-MisL-214	AAAAATACGATTGTCGTCGGC	SDM of p4403
Vf-MisL-229	ACTACCGGCGACAGTTCG	SDM of p4403
Vf-MisL-481	GATGATGATTCCGAAACGG	SDM of p4403
Vr-MisL-29	CCAGGCCGCCTGGGAGGG	SDM of p4403
Vr-MisL-128	CGCGGCATTCGCCGACTG	SDM of p4403
Vr-MisL-172	TCCGGCGTTAATACCGTC	SDM of p4403
Vr-MisL-282	CACCGTAATAGTAGCGTTG	SDM of p4403
Vf-MisL-671	GAGAAGAAGGTCACCAGC	SDM of p4403
Vf-MisL-476	GTGCTCAACGATGATGATTC	SDM of p4403
Vr-MisL-477	AGTATTAAAGACCAGCTTAC	SDM of p4403#

Vf-pWSK29	GAATTCCTGCAGCCCGGGG	GA of pWSK29 and p4251
Vr-pWSK29	AAGCTTATCGATACCGTCGACCTC	GA of pWSK29
Vr p4251	TTCACTTTTCTCTATCACTGATAGGGAGTGGTA	GA of p4251
1f PtetA-bapA	CCCTATCAGTGATAGAGAAAAGTGAATCCATCAGGAGCTGATTTATG	GA of p4251
1r-bapA-VCBN1-3	AGGTGGTAGGTGCCCGTCGGCGCGGTATCAT	GA of p4251
2f-bapA-VCBN1-3	CGGGCACCTACCACCTGTTAAACGGCGCGGAC	GA of p4251
2r bapA-end	CACCGCTTGTTGCCCCGAACGTC	GA of p4251
3f bapA-bapB	GACGTTCGGGGCAACAAGCGGTGATATTTAAAAGGGATAAAC	GA of p4251
3r-bapD-pWSK29	CCGGGCTGCAGGAATTCGCTAAAGCGTAGGTTTCGT	GA of p4251

Suppl. Figures and Legends

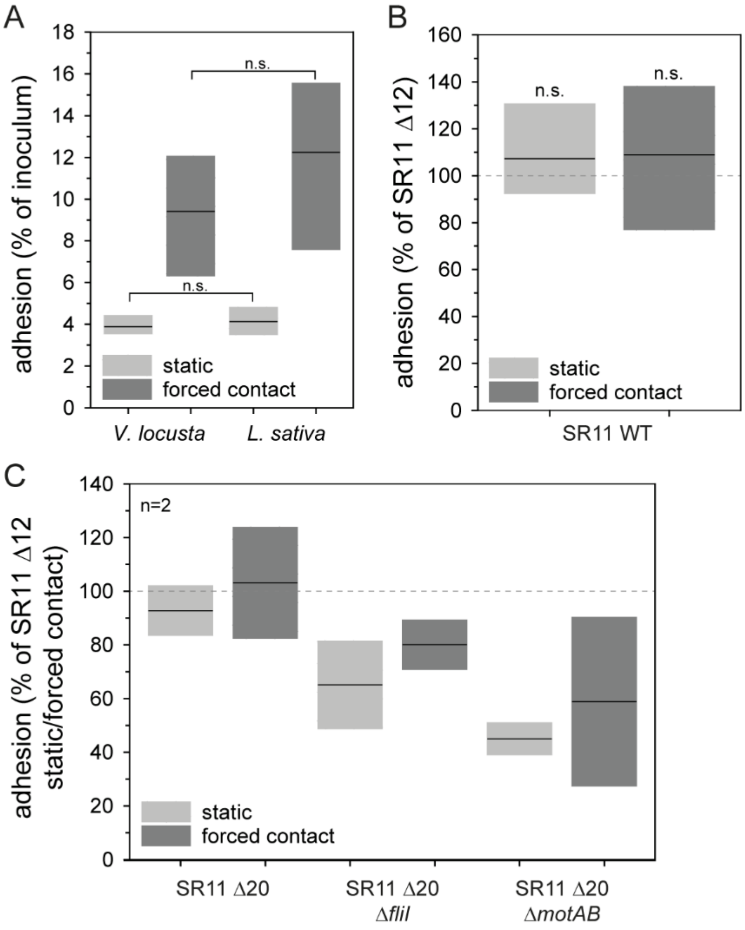

Fig. S 1. Comparison of *Salmonella* SR11 WT, SR11 Δ12 and SR11 Δ20 and SR11 Δ12 in adhesion to *V. locusta* and lettuce. A) Leaf discs obtained from *L. sativa* or *V. locusta* grown under aseptic conditions were inoculated with STM SR11 Δ12. The STM inoculum was applied without further treatment (static), or with centrifugation for 5 min at 500 x g to increase contact of STM to salad surface (forced contact). CFU counts of STM were quantified in inoculum and in plant tissue homogenates. Adhesion was calculated as percentage of inoculum bound to leaf discs. **B**) *L. sativa* leaf discs were inoculated with SR11 WT or SR11 Δ12. Inoculation was performed under static conditions or using forced contact, and CFU counts in inoculum and leaf homogenates were determined. Adhesion of SR11 Δ12 was normalized to 100% and adhesion of SR11 WT is shown as percentage of adhesion of SR11 Δ12. **C**) *L. sativa* leaf discs were inoculated with STM SR11 Δ20, or non-motile derivatives SR11 Δ20 Δ*fliI* or SR11 Δ20 Δ*motAB,* defective in flagella filament formation, or in energizing flagellar rotation, respectively. The inoculation was performed under static conditions, or by forced contact mediated by centrifugation at 500 x g for 5 min. Adhesion is expressed as percentage of adhesion of SR11 Δ12 normalized to 100%. The box plots show means of adhesion, medians, and the statistical significances were determined as described in **Fig. 2**.

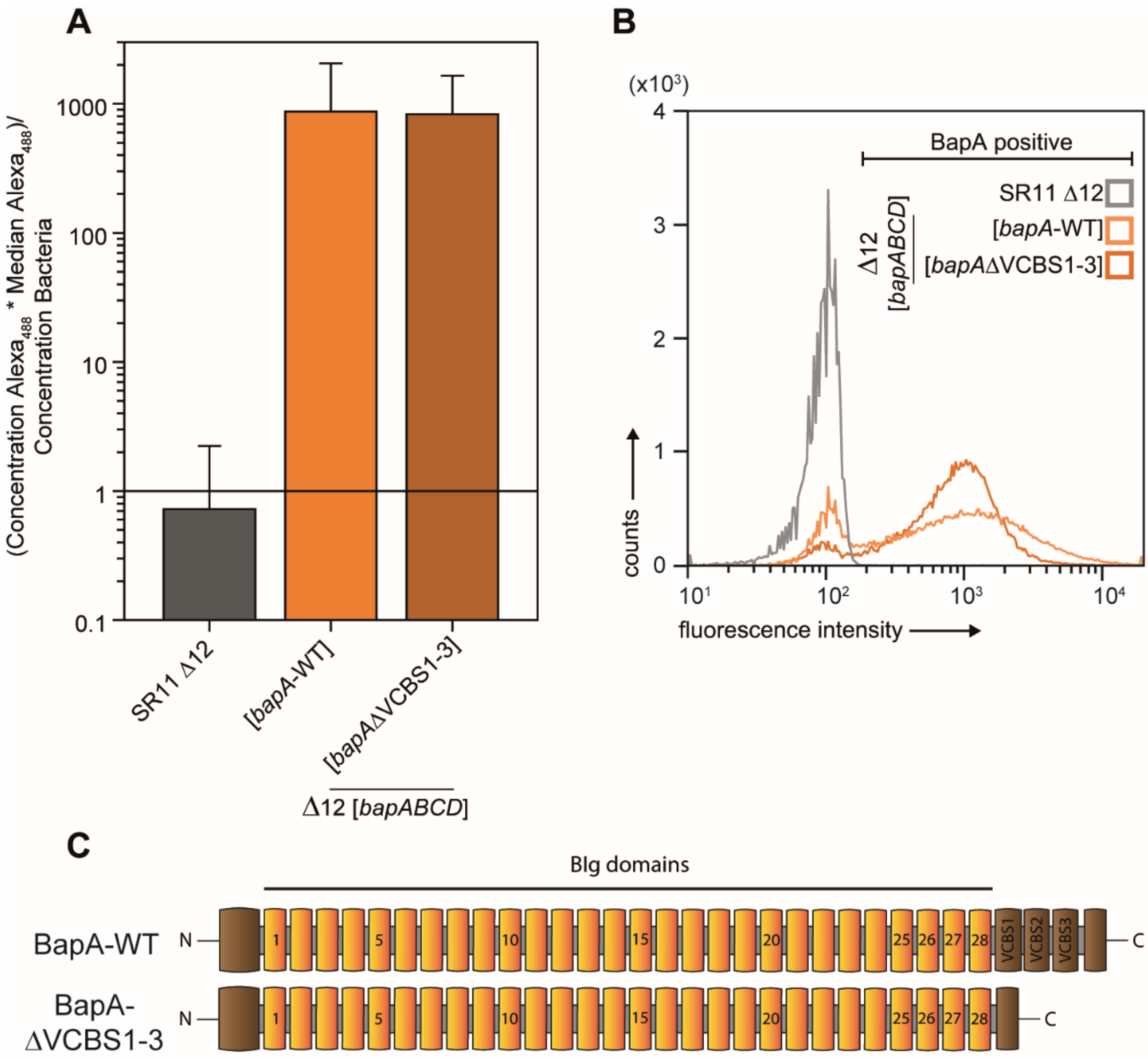

Fig. S 2. Quantification of BapA surface expression by flow cytometry. AHT-induced surface expression of adhesin BapA and mutant allele BapA-ΔVCBS1-3 was quantified by flow cytometry. BapA were detected using rabbit-α BapA (dilution 1:1,000), and goat-α rabbit-Alexa488 (dilution 1:2,000) as secondary antibody. **A**) Quantification of BapA-positive bacteria by flow cytometry. Data were normalized to concentration and median Alexa488 fluorescence and concentration of measured bacteria (n=3). **B**) Overlay of the measured fluorescence intensities of one representative experiment. **C**) Schematic overview of truncated form of BapA with deletion of VCBS1-3.

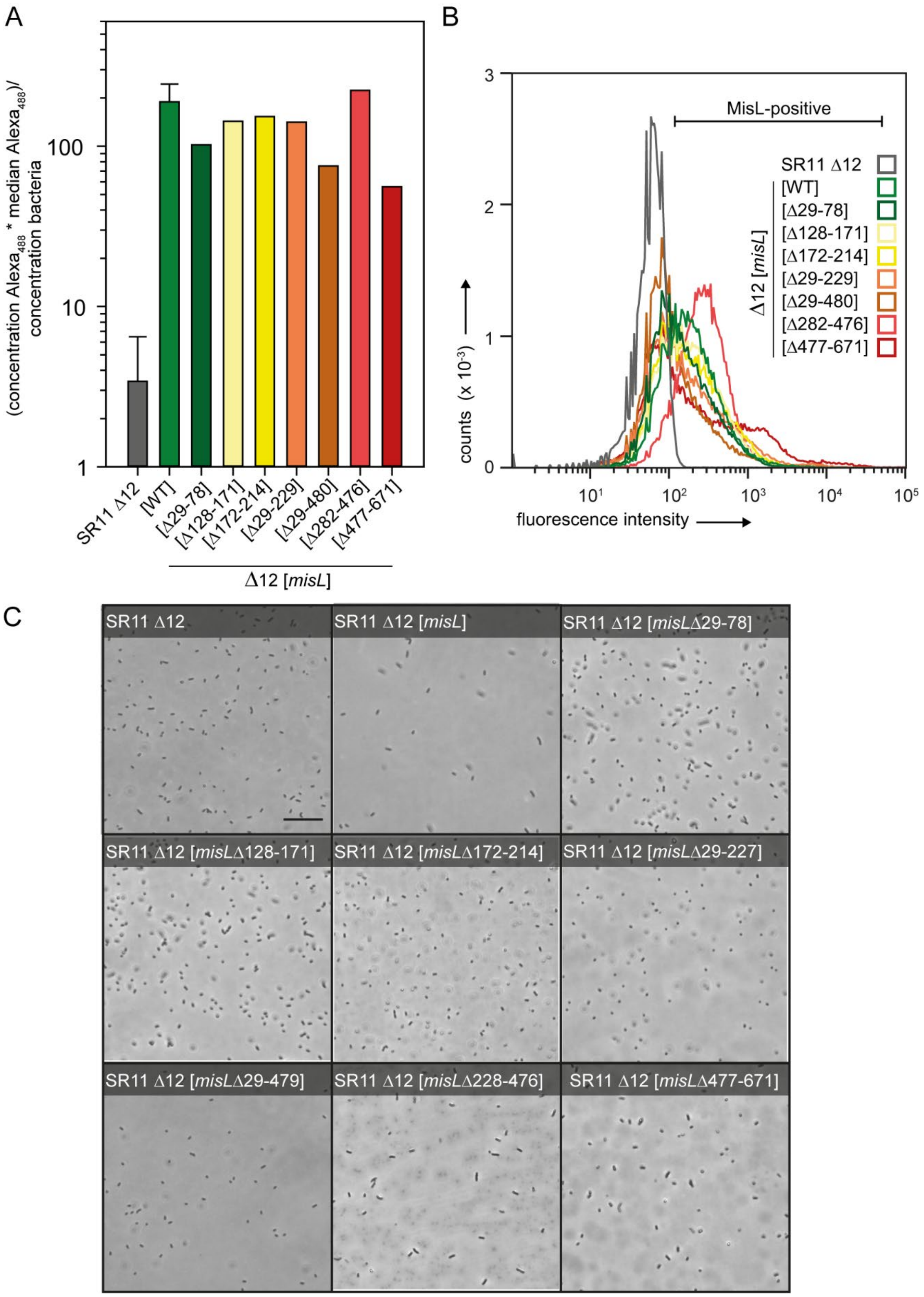

Fig. S 3. Quantification of MisL surface expression and analysis of bacterial autoaggregation. AHT-induced surface expression of MisL and various mutant alleles of MisL was quantified by flow cytometry. MisL was detected using rabbit α MisL (dilution 1:1,000), and goat α rabbit-Alexa488 (dilution 1:2,000) as secondary antibody. **A**) Flow cytometry data

of Alexa488-positive bacteria normalized to concentration and median Alexa488 fluorescence and concentration of measured bacteria. **B**) Overlay of fluorescence intensities from a representative analysis. **C**) Micrographs of various strains encoding WT MisL or truncated forms of MisL. Bacteria were grown in LB broth with and without AHT induction (+AHT, -AHT, respectively). Aliquots of cultures were spotted on microscopy slides and images were recorded using a Zeiss Axio Observer with AxioCam and further processed by ZEN 2012. Scale bar, 20 μm.

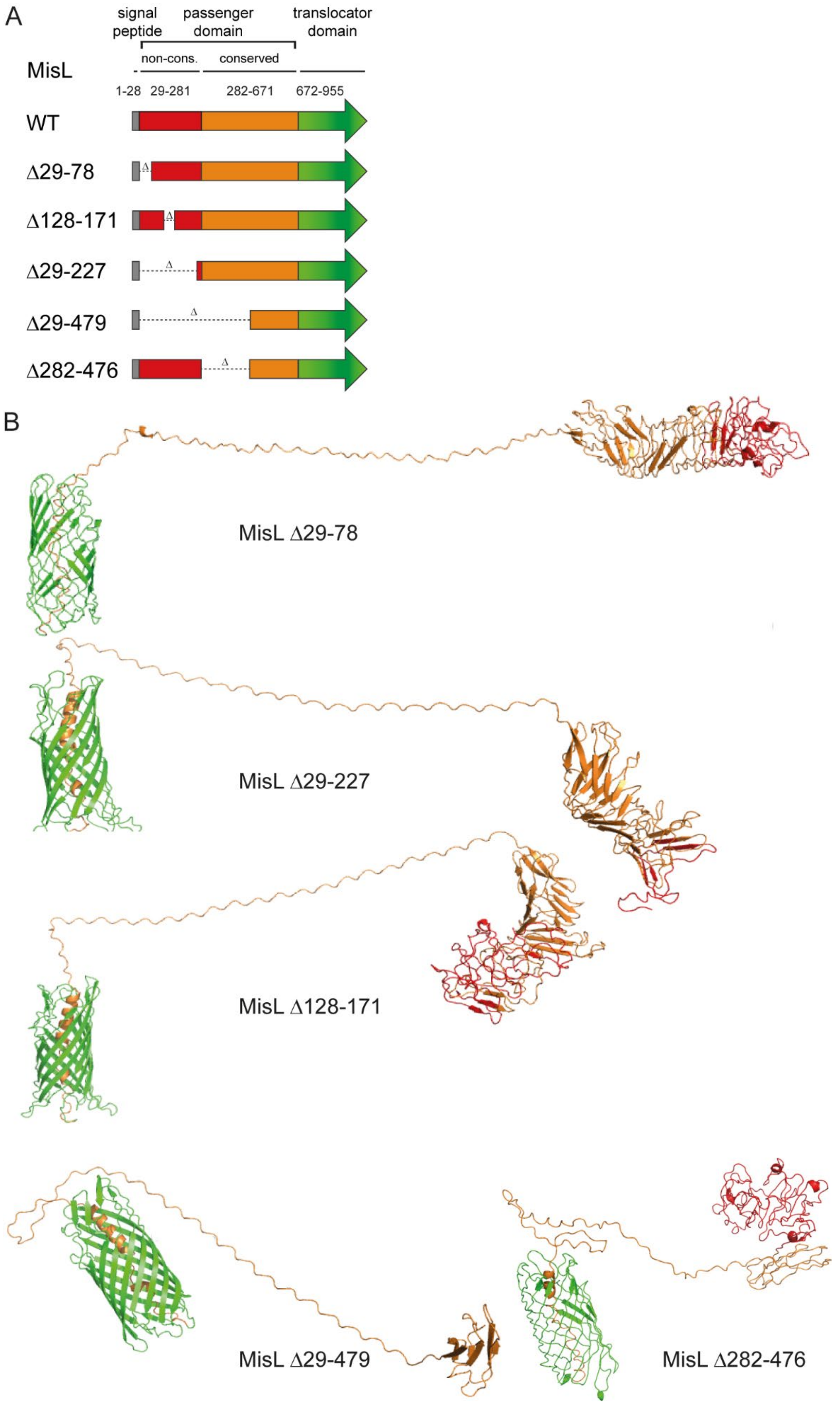

Fig. S 4. Homology-based protein structure prediction for truncated forms of MisL. Structure predictions were performed by trRosetta and visualized by PyMol. Colors represent

translocator domain (green), conserved passenger domain (orange) and non-conserved passenger domain (red). Tm scores of protein structure prediction of MisL and mutant alleles were MisL WT, 0.258; MisL Δ29-78, 0.293; MisL Δ128-171, 0.259; MisL Δ172-214, 0.26; MisL Δ29-227, 0.4; MisL Δ29-497, 0.665; MisL Δ282-476, 0.242; MisL Δ477-671, 0.333.
